# Utilization of local agro-industrial by-products based substrates to enhance production and dietary value of mushroom (*P. ostreatus*) in Ethiopia

**DOI:** 10.1007/s11274-024-04062-3

**Published:** 2024-07-22

**Authors:** Buzayehu Desisa, Diriba Muleta, Tatek Dejene, Mulissa Jida, Abayneh Goshu, Tadesse Negi, Pablo Martin-Pinto

**Affiliations:** 1 Institute of Biotechnology, Addis Ababa University, P.O. Box 1176, Addis Ababa, Ethiopia; 2 Ethiopian Forestry Development, P.O. Box 24536, Addis Ababa, 1000 Ethiopia; 3 Bio and Emerging technology Institute, P.O. Box 5954, Addis Ababa, Ethiopia; 4 Sustainable Forest Management Research Institute, University of Valladolid (Palencia), Avda, Madrid 44, Palencia, 34071 Spain; 5 Ethiopian Sugar Industry Group, Wonji, Ethiopia

**Keywords:** *Pleurotus ostreatus*, Agro-industrial byproducts, Yield, Biological efficiency, Food security

## Abstract

Food insecurity and malnutrition are serious problems in many developing countries, including Ethiopia. This situation warrants an urgent need for the diversification of food sources with enhanced productivity. This study was aimed at contributing to the food security in Ethiopia through cultivation of *Pleurotus ostreatus* mushrooms using sustainable and locally available agro-industrial byproduct-based substrates in parallel with pollution control. Ten substrates were prepared using sugarcane bagasse, filter cake, trash, cotton seed hull and animal waste, namely cow dung and horse and chicken manure. The effect of each substrate (treatment) on the yields, biological efficiency, nutritional composition, and mineral contents of *Pleurotus ostreatus* mushroom species was evaluated at the Ethiopian Forest Products Innovation Center, Addis Ababa, Ethiopia. The results obtained indicate that a significantly higher (*p* < 0.05) yield and biological efficiency were recorded from the mushroom cultivated on S2 substrate containing a mixture of 80% sugarcane bagasse, 12% cow dung, and 8% cotton seed hull. Moreover, substrate containing sugarcane bagasse mixed with cotton seed hull, cow dung, and chicken manure significantly (*p* < 0.05) increased the yields and biological efficiency of the mushroom. The content of protein, crude fat, fiber, and carbohydrates of the mushroom cultivated from all the utilized substrates were in the range of 17.30–21.5, 1.77–2.52, 31.03–34.38, and 28.02–39.74%, respectively. The critical macro-elements are abundant in the mushroom in the order of potassium, magnesium, calcium, and sodium. The mushrooms cultivated on all the substrates were rich in essential micro-elements in the order of iron and zinc. It was found that substrate preparation and formulation significantly (*p* < 0.05) improved the yields, biological efficiency, nutritive values, and mineral contents of the mushroom. The use of these by-products as substrates is sustainable and environmentally friendly and allows the production of mushroom with high nutritional value on a sustainable basis in order to enhance food security in the country.

## Introduction

In developing countries like Ethiopia, ensuring food security remains a major challenge. Despite the world’s rapid population growth, the prevalence of food insecurity remains a significant global issue. Recent data from the FAO (2022) reveals an alarming increase of 112 million people unable to afford a nutritious diet, totaling almost 3.1 billion worldwide. Factors such as pandemics, climate change, social unrest, escalating food prices, and high unemployment rates further exacerbate the food security crisis (Béné et al. 2019; FAO 2022). Addressing these challenges requires innovative solutions in agro-food systems, promoting diversification, and improving food production. Edible mushroom cultivation emerges as a promising biotechnological and climate-resilient approach to fulfill the demand for nutritious food with high biological and medicinal value, countering malnutrition. Additionally, enhancing agricultural sustainability involves recycling agro-industrial and forestry by-products, contributing to a circular economy (Kumar et al. 2021).

Mushrooms, known for their unique flavor, texture, and nutritional richness, offer a promising solution for ensuring food security and combating malnutrition in developing countries (Fernandes et al. 2021). In Ethiopia, mushroom cultivation could be highly attractive because of the favorable climates and huge accumulation of lignocellulosic biomass derived from industrial and agricultural sectors. Mushroom cultivation technology can be considered profitable considering shrinking agricultural lands, growing unemployment rates, and widely available agro-industrial byproducts. Furthermore, mushrooms are acknowledged for their nutritional benefits, serving as a valuable source of non-starchy carbohydrates, dietary fiber, proteins, amino acids, minerals, and vitamins (Chang and Miles 2004; Zied et al. 2017; Yao et al. 2019). They offer a useful alternative to meat in vegetarian diets, being low in calorific value (Elkanah et al. 2022) and fat content while remaining crucial for good health due to their low or no cholesterol content (Dawadi et al. 2022).

Lignocellulosic biomass has been considered a noticeable source of nutrients for the mushrooms’ growth (Ruiz-Rodriguez et al. 2010). *Pleurotus species* have been cultivated successfully on a variety of agro-industrial byproducts for carbon sources that include wheat straw, soybean (Kumla et al. 2020); palm oil waste, shafts, bunches, sawdust, cotton seed hulls (Onyeka and Okehie 2018; Muswati et al. 2021), rice straw, corn cobs and sugarcane bagasse (Aigbodion et al. 2010). Each mushroom species requires an optimal C/N ratio in the substrate for cultivation (Carrasco et al. 2018). Supplementing agro-industrial byproducts with some nitrogen-rich substrates could improve the nutritional contents, yield, biological efficiency, mycelium growth, and quality of the fruiting bodies of the cultivated mushrooms better than those grown on the main substrate alone (Adenipekun and Omolaso 2015). The cultivation of edible mushrooms might be the only current process that combines the production of protein-rich food with the reduction of environmental pollution (Sánchez 2010). *Pleurotus ostreatus* mushroom is commonly cultivated in many towns and cities in Ethiopia without optimizing growth substrates. This study focused on improving the nutritional composition of the mushroom fruiting bodies to be harvested with better biological efficiency. Hence, this study was undertaken to develop new substrates from various agro-industrial byproducts to enhance the growth performance, yield, and nutrient contents of *Pleurotus ostreatus* fruiting bodies.

## Materials and methods

### Experimental site and design

This experiment was conducted at the Forest Products Innovation Center, located in Addis Ababa. The mycelium of the *Pleurotus ostreatus* mushroom species was used as an inoculum. Different combinations of locally available agro-industrial and animal by-products, such as cow dung, horse and chicken manures, cotton seed hull, sugarcane filter cake, and sugarcane trash with sugarcane bagasse, were used as substrates. The experiment was arranged using a Completely Randomized Design (CRD) with ten treatments and three replications with a total of 30 treatments combination.

### Culture source and tissue culture preparation

**Fig. 1:**
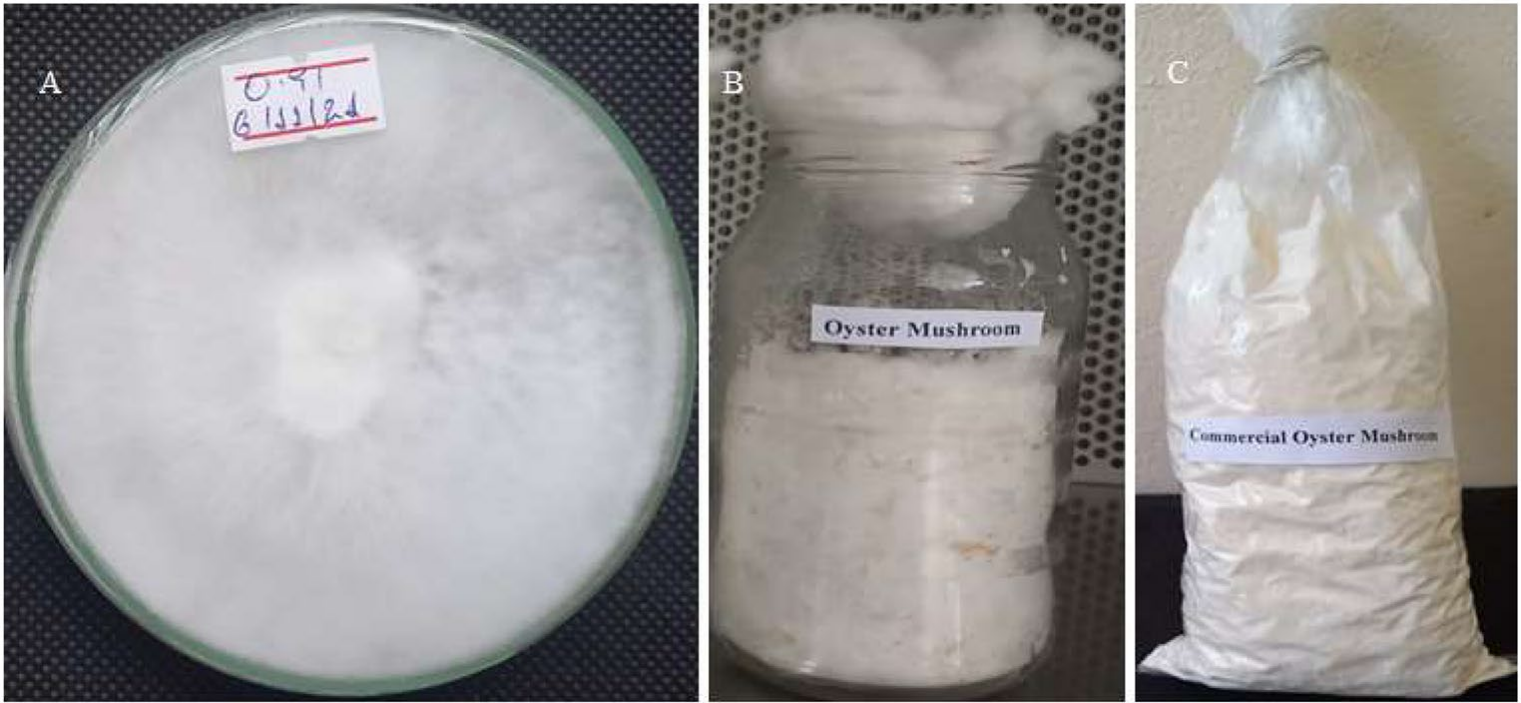
*P.ostreatus* mushroom mycelium growth on PDA (**A**), mother spawn (**B**), and commercial spawn (**C**)

*Pleurotus ostreatus* mushroom was obtained from the Mycology Laboratory, Department of Microbial, Cellular, and Molecular Biology, Addis Ababa University. The tissue culture of the mushroom was prepared on potato dextrose agar (PDA, HiMedia) medium and stored at 4 °C until required for use (Adebayo et al. 2013). A pure culture of *Pleurotus ostreatus* was used for spawn preparation (Fig. 1a).

### Grain mother and commercial spawn preparation

Wheat grains were weighed (375 g/bottle), washed, boiled for 45 min, and dried (Adebayo et al. 2013). The mother spawn was prepared in 500-mL glass jars filled with 95% wheat grain, 4% gypsum, and 1% calcium carbonate (Beje et al. 2013). The spawn substrate was packed in ¾ of the bottle and autoclaved at 121^o^C for 80 min (Atila 2017). After cooling at room temperature, the sterilized substrates were inoculated with 5 cm^2^ of actively growing mycelial agar discs (Yang et al. 2013). The inoculated jars were incubated for 10 days at a temperature of 25 ± 2°C until the mycelia fully covered the grains (Fig. 1b).

To propagate the commercial spawn of *P. ostreatus*, a polypropylene bag measuring 7.5 × 35 cm was filled with 15 g of grains on a w/w wet weight basis from mother spawns according to the method of Atila (2019), with some modifications. The prepared grains followed the same procedure as the mother spawn, and the mycelium fully covered the grains after 9 days (Fig. 1c).

### Collection, preparation, and formulation of the substrates

The growth substrates of sugarcane byproducts such as sugarcane bagasse, trash, and filter cake were collected from the Wonji sugar factory. Cow dung, chicken and horse manures, and cotton seed hull were obtained from Bishoftu City, Oromia Regional State, and used as supplements or ingredients. The seven growth substrates were completely dried under the sun and chopped into 4 cm pieces according to the method of Yang et al. (2013). Ten different formulations (treatments) of the agro-industrial byproducts and supplement-based substrates (S1–S10) were prepared from different ingredients. Sugarcane bagasse was used as the main substrate, weighed separately, and thoroughly mixed manually according to the proportion of the substrates as indicated in Table 1.

**Table 1:** Substrate formulations for the cultivation of *P. ostreatus* mushroom

Substrates/Treatments)	Compositions (Formulation)
S1	80% Sugar cane bagasse + 12% chicken manure + 8% cotton seed hull
S2	80% Sugar cane bagasse + 12% cow dung + 8% cotton seed hull
S3	80% Sugar cane bagasse + 12% horse manure + 8% cotton seed hull
S4	80% Sugarcane bagasse + 6.67% cow dung + 6.67% horse manure + 6.67% cotton seed hull
S5	80% Sugarcane bagasse + 20% cotton seed hull
S6	50% Sugarcane bagasse + 15% sugarcane trash + 15 sugarcane filter cake + 10% chicken manure + 10% cotton seed hull
S7	50% Sugarcane bagasse + 15% sugarcane trash + 15 sugarcane filter cake + 10% cow dung + 10% cotton seed hull
S8	50% Sugarcane bagasse + 15% sugarcane trash + 15 sugarcane filter cake + 10% horse manure + 10% cotton seed hull
S9	50% Sugarcane bagasse + 15% sugarcane trash + 15% sugarcane filter cake + 10% cow dung + 10% horse manure
S10	100% Sugarcane bagasse

All the treatments (substrates) were prepared from sustainable and locally available ingredients. The mixed substrates (500 g dry weight) were soaked in water for 24 h, and excess water was drained to adjust the moisture content to 60–65%. About 1% calcium carbonate and 1% gypsum (on a dry weight basis) were added to each substrate. The final substrates were filled in a polypropylene bag (7.5 × 35 cm) and autoclaved at 121 °C for 80 min.

### Analysis of proximate composition of the formulated substrates

The content of moisture and total ash were determined according to the Ethiopian Conformity Assessment Enterprise test (ES1032-1:2005) adopted from AOAC (1984) and AOAC (1995), respectively. Lignocellulose contents such as alcohol-toluene solubility and Klason lignin, hemicellulose and cellulose contents of the substrates were measured by ASTM D 1107-56, through direct extraction with aqueous alkali and Kurchner-Hoffer methods, respectively (Sánchez et al. 2014). Total crude fiber was determined by BCTL/SOP/M017.01 adopted from ISO 5498, agricultural food product analysis manual (Indian Standard (ISO 5498 − 1981) 2005). The Soxhlet extraction technique described by Srigley and Mossoba (2016) was used to determine the crude fat (BCTL/SOP/M001.01) and crude proteins (BCTL/SOP/M014.01) content of the substrates. The N content of the substrates was measured by Kjeldahl method. The carbon content was obtained from a combination of the fixed carbon, volatile matter, and ash content of the biomass as suggested by (Lu et al. 2019). The Carbon-to-Nitrogen Ratio was quantified as C/N ratio using the following formula (Fauzan et al. 2022).

$$\text{C/N} \,Ration\, of\, Raw\, material=\frac{\%C}{\%N}$$

The concentrations of macro (K, Ca, Mg and Na) and microelements (Fe, and Zn) in the substrates were quantified by Microwave Plasma Atomic Emission Spectroscopy (MP-AES, Malaysia).

### Inoculation and incubation

The sterilized substrates, cooled at room temperature, were inoculated with 15 g of oyster mushroom spawn. The sterilized substrates in each bag were separately inoculated with three layers of 5 g of oyster mushroom spawn per layer on the bottom, middle, and surface. A total of thirty replicated polyethylene bags, ten from each of the treatments, were used for each substrate. The inoculated bags were placed in a spawn running room where light, temperature, and relative ambient humidity were controlled. The inoculated substrates were kept in a spawn running room at 25 °C and 70% relative humidity under dark conditions. The spawn run period to total colonization (the number of days from inoculation to complete colonization of the substrate by the mycelium) was recorded.

### Harvesting and determination of biological efficiency

After the entire substrate block was covered with mycelia, the maturation phase was induced using light, air, temperature, and the relative ambient humidity of the room, which were maintained at about 18–22 °C and 85–90%, respectively. The substrate bags were opened, and after 3–5 days, pinheads started to appear on the substrate in each polyethylene bag. The cropping room was watered intermittently to maintain moisture during the cropping time. After 2–3 weeks, the first flushes of mushroom fruit bodies started to develop and were harvested. Evaluation of cultivation and fruiting parameters such as time (days) of the first appearance of pinhead formation, time to first harvest (days), weight for three flushes (g), yield (g/kg), and biological efficiency (%) were measured (Iqbal et al. 2005; Yang et al. 2013). Yields were obtained from three to six flushes and expressed as grams of fresh mushrooms harvested at maturity per gram of dry substrates (w/w), and biological efficiency was calculated as a percentage ratio of the fresh weight of harvest per gram of dry substrates (Atila 2019).

### Analysis of proximate composition of mushroom fruiting bodies

All the harvested mushroom fruiting bodies were collected and treated as individual samples from each substrate (S1-S10). Samples used for analyses of nutritional composition were oven dried at 45 °C for 72 h, and ground to pass through sieve size of < 1 mm. The mushroom samples were analyzed for moisture (dry matter), ash, protein, fat, fiber, carbohydrates, and mineral elements (Mg, K, Ca, Na, Zn and Fe) at Ethiopian Conformity Assessment, chemical testing laboratory following standard methods.

Total ash and moisture contents in mushrooms were determined according to the Ethiopian Conformity Assessment Enterprise test (ES1032-1:2005) using an Agilent 4200 Series MP-AES Inductively Coupled Plasma spectrophotometer (Agilent Technologies, Santa Clara, CA, USA). This is adopted from the Association of Official Analytical Chemists AOAC (1984) for ash, and AOAC (1995) for moisture content, respectively. The crude protein content (*N*×6.25) of the samples was estimated by the micro-Kjeldahl method according to the method of BCTL/SOP/M014.01 adopted from USDA (2009) national nutrient database.

The total crude fiber was determined by BCTL/SOP/M017.01 adopted from ISO 5498, agricultural food product analysis manual (Indian Standard (ISO 5498 − 1981) 2005). The Soxhlet extraction technique described by Srigley and Mossoba (2016) was used to determine the crude fat (BCTL/SOP/M001.01). The available carbohydrate contents were determined according to the method of Teke et al. (2020). Available carbohydrate = 100- total protein + total lipid + crude fiber + ash. The analysis of main macro elements (Ca, Mg, K, and Na) and microelements (Fe and Zn) was quantified according to EPA 6020B, Modified Microwave Plasma Atomic Emission Spectroscopy (MP-AES).

### Statistical analysis

To assess the impact of different substrates on the growth and yield of *Pleurotus ostreatus* mushrooms, comparisons were made in terms of variables related to spawning time, days to pinhead formation, days to first harvest, yield, and biological efficiency. Data analyses were performed using Statistical Package for Social Sciences (SPSS) version 26. Data were log-transformed when needed to achieve the parametric criteria of normality and homoscedasticity necessary for the analysis of variance. Differences between substrate options for the different variables were evaluated using a one-way analysis of variance. Duncan’s Multiple Range Test (DMRT) was used to check for significant differences (*p* ≤ 0.05) between substrates when needed.

## Results

### The carbon/nitrogen ratio and lignocellulosic content of the substrates

The carbon/nitrogen (C/N) ratio of the mushroom growth substrates varied significantly (*p* < 0.05) among the formulated substrates (Table 2), and it is an important factor in *P. ostreatus* mushroom cultivation. Significantly higher and lower (*p* < 0.05) carbon values of 49.71% and 39.93% were obtained from S5 substrates and S9 substrates, respectively. A significantly higher (*p* < 0.05) nitrogen content of 1.59% was recorded from the S1 substrate, while a relatively lower value of nitrogen (0.51%) was obtained from the S10 substrate (control treatment) compared to the others. The C/N ratio of 29.24:1 was calculated for the S1 substrate, the value of which was lower than that of the other substrates. The result showed that a significantly higher (*P* < 0.05) C/N ratio of 90.37:1 was obtained from the S10 substrate. The highest (40.23%) and lowest (26.71%) cellulose contents were reported for S10 substrate and S9 substrate, respectively. The highest content (26.72%) of hemicellulose was recorded from the S1 substrate, while the lowest (9.21%) was obtained from the S3 substrate. On the other side, the highest (26.09%) and lowest (16.96%) lignin content were obtained from S2 and S10 substrates, respectively. In summary, there were significant differences (*p* < 0.05) between the substrates (treatments) in mean lignocellulosic content.

**Table 2:** C/N ratio and lignocellulosic contents of the growth substrate used in the study

Ratio of carbon to nitrogen				Lignocellulosic contents (wt%)		
Substrates	C (%)	N (%)	C/N (%)	Cellulose	Hemicellulose	Lignin
S1	46.51 ± 0.57^b^	1.59 ± 0.01^a^	29.24 ± 0.40^c^	32.12 ± 1.05^de^	26.72 ± 0.53^a^	18.49 ± 0.90^g^
S2	44.73 ± 0.05^c^	1.10 ± 0.01^e^	40.42 ± 0.18^bc^	31.72 ± 1.55^f^	17.86 ± 0.61^c^	26.09 ± 0.42^a^
S3	44.49 ± 0.43^c^	0.94 ± 0.02^g^	47.51 ± 0.19^bc^	37.22 ± 0.66^b^	9.21 ± 1.53^h^	22.54 ± 0.10^d^
S4	42.33 ± 0.02^e^	1.22 ± 0.04^d^	34.60 ± 0.14^bc^	33.32 ± 0.90^cd^	13.83 ± 0.06^f^	25.88 ± 0.28^a^
S5	49.71 ± 0.34^a^	1.22 ± 0.20^d^	40.46 ± 0.53^bc^	37.24 ± 0.21^b^	22.21 ± 0.08^b^	21.27 ± 0.33^e^
S6	44.34 ± 0.01^c^	1.39 ± 0.01^b^	50.98 ± 31.13^b^	34.25 ± 0.06^c^	21.41 ± 0.25^b^	19.30 ± 0.32^f^
S7	43.50 ± 0.01^d^	1.08 ± 0.11^e^	40.15 ± 0.42^bc^	34.29 ± 0.24^c^	16.27 ± 0.13^d^	24.29 ± 0.23^b^
S8	43.51 ± 0.01^d^	0.99 ± 0.12^f^	43.84 ± 0.14^bc^	38.23 ± 0.11^b^	15.21 ± 0.01^e^	21.32 ± 0.37^e^
S9	39.93 ± 0.05^f^	1.27 ± 0.06^c^	31.28 ± 0.13^c^	26.71 ± 0.57^f^	11.30 ± 0.05^g^	23.32 ± 0.38^c^
S10	46.08 ± 0.01^b^	0.51 ± 0.01^h^	90.37 ± 1.78^a^	40.23 ± 1.28^a^	17.68 ± 0.11^c^	16.96 ± 0.23^h^

Values in parenthesis are the standard deviation. Values in the same column with different letters as superscripts are significantly different (*p* < 0.05). For S1-S10, see Table 1

### Nutritional composition of the substrates

The nutritional compositions of the substrates for the highest growth of *P. ostreatus* mushrooms are listed in Table 2. Different substrate formulations affected the percentage of crude protein in the growth substrate of oyster mushrooms. The crude protein content of the substrates obtained in this study was significantly (*p* < 0.05) different among the substrates formulated from different ingredients (Table 3) and ranged from 3.41 to 10.32%. The highest significant protein content was obtained from the S1 substrate. However, the lowest significant crude protein was obtained from the S10 substrate (sugarcane bagasse alone). The crude fiber content of the substrates ranged between 23.16% and 37.15% and was significantly higher (*p* < 0.05) for S10, the control substrate, compared to that of the others. The maximum and minimum carbohydrate contents of 53.8% and 41.34% were obtained from S5 and S10 substrates, respectively (Table 3).

**Table 3:** Nutritional composition of the substrates used for the growth of *P. ostreatus* mushrooms

Substrates	Nutritional composition (% on dry basis)			
	Crude protein	Crude fat	Crude fiber	Carbohydrate
S1	10.32 ± 0.27^a^	1.35 ± 0.20^c^	24.25 ± 0.05^f^	47.58 ± 0.82^c^
S2	7.34 ± 0.01^d^	1.32 ± 0.01^c^	26.52 ± 0.09^e^	46.45 ± 0.46^d^
S3	6.21 ± 0.03^f^	1.22 ± 0.11^c^	29.48 ± 0.01^b^	45.45 ± 0.74^d^
S4	8.12 ± 0.02^c^	1.29 ± 0.05^c^	27.62 ± 0.06^d^	42.34 ± 0.83^e^
S5	4.12 ± 0.01^g^	1.24 ± 0.39^c^	28.44 ± 0.57^c^	53.84 ± 1.21^a^
S6	9.18 ± 0.06^b^	1.95 ± 0.05^a^	23.16 ± 0.02^g^	49.35 ± 0.15^b^
S7	6.21 ± 0.10^f^	1.88 ± 0.01^a^	24.48 ± 0.12^f^	50.11 ± 0.19^b^
S8	7.09 ± 0.10^e^	2.08 ± 0.01^a^	26.37 ± 0.06^e^	48.24 ± 0.52^c^
S9	8.15 ± 0.05^c^	1.82 ± 0.03^ab^	26.62 ± 0.16^e^	41.85 ± 0.50^e^
S10	3.41 ± 0.01^h^	1.61 ± 0.08^b^	37.15 ± 0.07^a^	41.34 ± 0.23^e^

Legend: Different lowercase letters denote significant differences in each column (*p* < 0.05). For S1-S10, see Table 1

### Mineral composition of the substrates

As indicated in Table 4, the S1 substrate had a significantly higher (*p* < 0.05) potassium content of 11,172 mg/kg compared to all the others, while the S10 substrate had a lower potassium content of 3592 mg/kg. The mean content of zinc and sodium was also significantly higher (*p* < 0.05) for the S1 substrate compared to the others, whereas relatively lower contents of zinc and sodium were recorded for the S10 and S5 substrates, respectively (Table 4). The highest iron (Fe) and magnesium (Mg) concentrations (7773 and 3285 mg/kg) were recorded from the S9 substrate, while the S5 substrate contained the lowest contents of Fe (1062 mg/kg) and Mg (874 mg/kg), respectively. Substrate S7 contained the highest calcium content (14,002 mg/kg), followed by S9 (13,718 mg/kg) and S2 (12,352 mg/kg), while S10 substrate recorded the least calcium content (1040 mg/kg). As a result, the formulations affected the mineral compositions of the substrates (Table 4). Therefore, there were significant mineral contents in each prepared mushroom substrate used for the cultivation of *P. ostreatus* mushrooms.

**Table 4:** Mineral composition of the growth substrate formulas

Substrate	Minerals (mg/kg)					
	Zn	Fe	Na	Mg	K	Ca
S1	43 ± 0.02^a^	2431 ± 0.05^c^	1630 ± 0.40^a^	2279 ± 0.05^d^	11,172 ± 0.57^a^	5103 ± 0.28^h^
S2	28 ± 0.11^d^	1134 ± 0.13^h^	1173 ± 0.13^e^	2205 ± 0.38^e^	8317 ± 0.28^b^	12,351 ± 0.10^c^
S3	18 ± 0.02^f^	1714 ± 0.07^f^	1020 ± 0.32^h^	1495 ± 0.94^h^	9040 ± 0.17^b^	5766 ± 0.22^g^
S4	23 ± 0.15^e^	1605 ± 0.07^g^	1280 ± 0.42^c^	2339 ± 0.05^c^	9445 ± 0.32^b^	11,118 ± .34^d^
S5	12 ± 0.10^g^	1061 ± 0.07^i^	830 ± 0.32^g^	873 ± 0.15^i^	7145 ± 0.18^b^	4940 ± 0.13^i^
S6	37 ± 2.24^b^	2539 ± 0.32^b^	1299 ± 0.25^b^	2041 ± 0.01^f^	9877 ± 0.19^b^	9653 ± 0.48^f^
S7	28 ± 0.49^d^	2191 ± 0.33^d^	1025 ± 0.21^g^	1961 ± 0.58^g^	8164 ± 0.89^b^	14,002 ± 0.27^a^
S8	22 ± 0.03^e^	2539 ± 0.34^b^	933 ± 0.33^i^	2535 ± 0.34^b^	8597 ± 0. 98^b^	10,051 ± 0.06^e^
S9	30 ± 0.05^c^	7773 ± 0.34^a^	1265 ± 0.24^d^	3285 ± 0.01^a^	8590 ± 0.18^b^	13,718 ± 0.06^b^
S10	8 ± 0.36^h^	2010 ± 0.15^e^	1160 ± 0.11^f^	2027 ± 53^e^	3591 ± 0.56^b^	1040.±0.19^j^

Legend: Different lowercase letters denote significant differences in each column (*p* < 0.05). For S1-S10, see Table 1

### Spawn running, pinhead formation and fruiting body formation

**Fig. 2:**
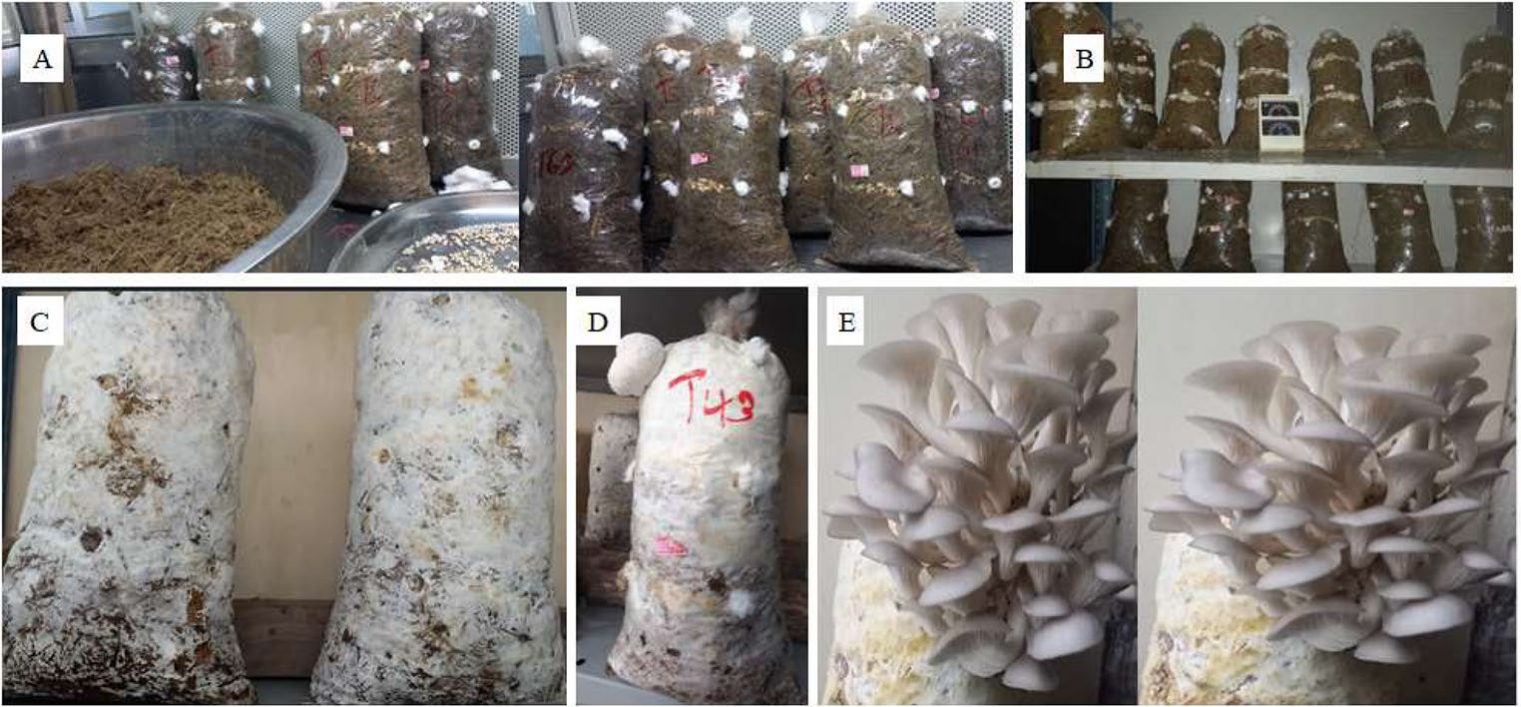
Substrate inoculation (**A**); spawn running (**B**); completion of spawn (**C**); pinhead formation (**D**); Matured fruiting bodies ready for harvest (**E**)

The time of mycelium colonization of the ten substrates by *P. ostreatus* was significantly (*p* < 0.05) different (Table 5). The colonization of the substrates was completed between 10 and 17.33 days. Among the different and potential substrates evaluated in the current study for the cultivation of *P. ostreatus* mushroom, the S7 substrate supported colonization within a short time compared to the others (Fig. 2a). The *P. ostreatus* assigned to S4 and S1 substrates required a relatively longer time of 17 and 16 days of complete colonization, respectively, compared to the others. There was a significant (*p* < 0.05) difference between the growth substrates in supporting the first *P. ostreatus* primordial appearance (Fig. 2b and c). Sugarcane bagasse alone or with supplements was found to be a better substrate for supporting pinhead formation by *P. ostreatus* (Table 5). The time taken for the first appearance of pinhead after the spawning of *P. ostreatus* assigned to different substrates varied from 3.33 to 6.67 days. Pinhead formation of *P. ostreatus* mushroom occurred earlier on the S1 substrate, followed by the S4 and S7 substrates. On the contrary, *P. ostreatus* assigned to S6 and S8 substrates exhibited an extended period for the formation of pinheads. As shown in Table 6, the first fructification was exhibited by *P. ostreatus* assigned to the S1 substrate within 2.33 days.

A relatively longer time for the first fructification of 5.33 days was recorded from *P. ostreatus* placed on the S7 substrate after the appearance of pinheads (Fig. 2de). The fastest mycelial growth was observed from *P. ostreatus* assigned to the S7 substrate, but this did not correspond with the first appearance of the fruiting bodies of mushrooms. The first appearance of pinheads on S1 substrates was initiated to produce the first fruiting bodies and was not related to the maturity of the fruiting bodies to be harvested (Table 5). This implies that the formula employed for the substrates’ formulation highly influences the product’s yield and quality (Table 1; Fig. 2).

A significant (*p* < 0.05) difference was observed among *P. ostreatus* assigned to different substrates in the mean number of days in the cropping chamber from the first harvest (Table 5). A significantly shorter (*P* < 0.05) first harvest of 3.30 days was recorded from *P. ostreatus* mushrooms subjected to S1 substrate, while the longest time for the first harvest of *P. ostreatus* of 7.33 and 9.33 days was obtained from S6 and S10 substrates, respectively. The C/N ratio had more effects on mycelium growth and the formulation and development of fruiting bodies (Fig. 2e).

### Cap diameter and stipe length

The results of the physical characteristics of *P. ostreatus* cultivated on different substrates are shown in Table 5. There was no significant difference (*p* > 0.05) between the *P. ostreatus* mushrooms cultivated on different substrates in the mean diameters of the cap and length of the stipe (Table 6). The experimental mushrooms grown on the S8 substrate had a wider cap diameter of 8.50 cm, followed by that of the S2 substrate with a cap diameter of 8.33 cm. The longest (7 cm) and shortest (5.05 cm) stipe lengths of fruiting bodies were recorded from *P. ostreatus* mushrooms cultivated on S7 and S1 substrates, respectively. The addition of supplements to the main substrate had no significant impact (*p* > 0.05) on cap diameter or stalk length (Table 5). However, S2 and S8 substrates are more suitable substrates for the cultivation of oyster mushrooms due to their bigger caps and relatively short stalk fruiting bodies.

**Table 5:** Effect of different substrate formulas on the morphological characteristics of the fruiting bodies

Substrates	Spawn run (days)	Pinhead formation (days)	Fructification (days)	First harvest (day)	Cap diameter (cm)	Stipe length (cm)
S1	17.11 ± 3.01^a^	3.33 ± 0.57^b^	2.33 ± 0.57^b^	3.30 ± 1.02^b^	6.66 ± 1.04^ab^	5.05 ± 0.76^b^
S2	16.33 ± 1.52^b^	4.00 ± 1.00^ab^	3.33 ± 0.57^ab^	3.67 ± 0.57^ab^	8.33 ± 0.76^a^	5.83 ± 1.04^ab^
S3	13.67 ± 2.51^d^	5.00 ± 0.10^ab^	2.67 ± 1.15^b^	4.67 ± 0.61^ab^	6.66 ± 1.25^ab^	6.66 ± 1.25^ab^
S4	14.33 ± 3.05c	3.67 ± 0.57^b^	4.33 ± 0.57^ab^	5.33 ± 1.52^ab^	7.22 ± 1.12^ab^	6.03 ± 0.50^ab^
S5	13.33 ± 1.52^d^	4.33 ± 1.15^ab^	3.67 ± 2.30^ab^	5.33 ± 1.50^ab^	7.16 ± 1.60^ab^	6.20 ± 1.12^ab^
S6	12.33 ± 3.23^e^	6.67 ± 2.88^a^	3.33 ± 1.15^ab^	7.33 ± 2.08^ab^	7.16 ± 0.28^ab^	5.04 ± 0.50^b^
S7	10.12 ± 0.01^c^	3.67 ± 1.15^b^	5.33 ± 0.57^a^	4.00 ± 3.03^ab^	6.66 ± 1.75^ab^	7.00 ± 1.05^a^
S8	13.00 ± 1.73^d^	6.00 ± 2.64^ab^	3.00 ± 1.01^b^	3.67 ± 1.09^b^	8.50 ± 0.86^a^	5.20 ± 0.34^ab^
S9	15.33 ± 4.50^bc^	4.00 ± 1.00^ab^	2.67 ± 1.52^b^	5.33 ± 1.52^ab^	6.66 ± 1.15^ab^	5.83 ± 0.76^ab^
S10	14.00 ± 1.73^c^	4.33 ± 0.57^ab^	2.67 ± 0.57^b^	9.00 ± 1.73^a^	5.83 ± 0.28^b^	6.10 ± 1.76^ab^

Different lower case letters denote significant differences in each column (*p* < 0.05). For S1-S10, see Table 1

### Weight, total yield, and biological efficiency

**Fig. 3:**
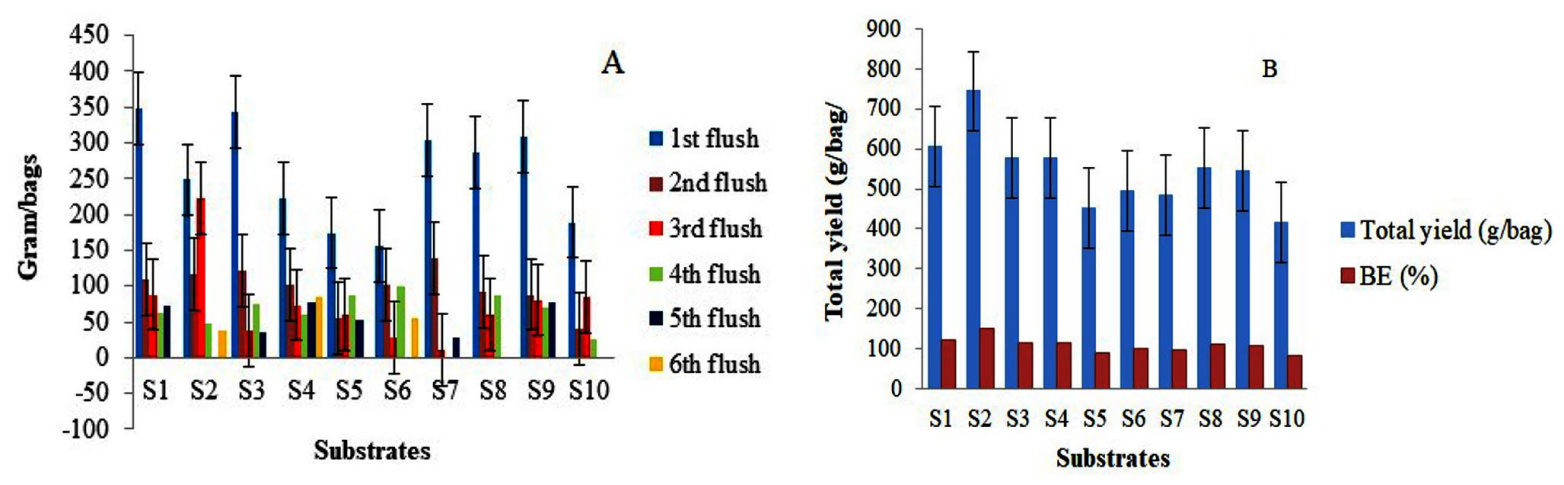
The effects of different substrate formulas on the weight of flush (**A**), yield, and B.E (%) (**B**) *P. ostreatus* mushrooms. The data are the mean results ± standard deviation. For S1-S10, see Table 1

The results of total fresh mushroom production and biological efficiency are shown in Fig. 3b. With regards to yield distribution among flushes, 3–6 flushes were harvested during the 10–15 days cycle, and over 90% of the total yield was obtained from the first and second flushes, followed by gradual decreases for all the substrates (Fig. 3a). The total yield harvested from the *P. ostreatus* mushroom cultivated on S2 substrate appeared to support the production of better mushrooms compared to all the others. The highest total mushroom yield of 744.20 g/500 g of the substrate was obtained from *P. ostreatus* mushroom assigned to the S2 substrate, followed by that of the S1 substrate (607.10 g/500 g), while *P. ostreatus* cultivated on the S5 substrate supported the lowest yield of total mushroom harvested (415.66 g/500 g; Fig. 3b). Sugarcane bagasse supplemented with chicken manure, cow dung, and cotton seed hull supported a higher yield than that of the control substrates (S10; 100% sugarcane bagasse). Figure 4 shows the biological efficiency of the substrates for their utilization and the amount of mushrooms produced. The productivity of *P. ostreatus* cultivated on. S2 and S1 substrates also showed significantly (*p* < 0.05) higher biological efficiency than that of *P. ostreatus* cultivated on the S10 substrate, which was the least efficient. The general tendency is that all the substrates supported high yields with higher biological efficiency.

The highest biological efficiency (B.E) of *P. ostreatus* mushrooms obtained from the S2 substrate was 148.84%, followed by S1 at 121% (Fig. 3b), whereas mushrooms cultivated on the S10 substrate had the lowest yield biological efficiency (83%). A substrate prepared using sugarcane bagasse with chicken manure, cow’s dung, and cotton seed hull had the best productivity and biological efficiency. On the contrary, the productivity and biological efficiency of mushrooms cultivated on sugarcane bagasse substrate alone had the lowest biological efficiency and productivity. All the other substrates were appropriate for the cultivation of *P. ostreatus* in terms of mushroom yield and biological efficiency (Fig. 3).

### Nutrient contents and fruiting bodies

The results of the nutritional composition of the *P. ostreatus* fruit bodies grown on the studied substrates are shown in Table 6. Variations in substrates and supplements significantly (*P* < 0.05) affected the nutrient content of oyster mushrooms. Thus, the highest crude protein (21.53%), crude fat (2.48%), and ash (6.95%) contents were recorded from *P. ostreatus* grown on S1 substrate, while the highest crude fiber (34.38%) and total carbohydrate (41.15%) contents were recorded from *P. ostreatus* cultivated on S4 and S10 substrate, respectively. The highest nutrient contents were recorded from the mushrooms cultivated on supplemented substrates as compared to non-supplemented substrates, except those cultivated on S10 and S4 substrates.

**Table 6:** Nutritional value of *Pleurotus ostreatus* cultivated on different substrates (% by mass)

Substrates	Crude protein	Carbohydrate	Crude fiber	Crude fat	Moisture	Ash
S1	21.53 ± 0.25^a^	28.02 ± 0.22^f^	33.47 ± 0.01^ab^	2.48 ± 0.05^a^	7.55 ± 0.01^b^	6.95 ± 0.04^a^
S2	20.36 ± 0.15^b^	32.61 ± 0.19^d^	32.66 ± 0.01^abc^	2.11 ± 0.05^b^	5.52 ± 0.06^e^	6.74 ± 0.06^fg^
S3	19.55 ± 0.05^c^	30.78 ± 0.01^e^	33.65 ± 0.01^ab^	1.88 ± 0.01^c^	6.76 ± 0.02^c^	7.38 ± 0.05^b^
S4	19.43 ± 0.01^c^	30.16 ± 0.04^e^	34.38 ± 0.04^a^	2.13 ± 0.05^b^	6.45 ± 0.05^c^	7.45 ± 0.05^c^
S5	20.46 ± 0.05^b^	31.65 ± 0.05^d^	31.70 ± 0.02^bc^	2.52 ± 0.01^a^	8.22 ± 0.03^a^	5.45 ± 0.04^g^
S6	20.50 ± 0.10^b^	31.70 ± 0.09^d^	32.33 ± 0.01^abc^	2.13 ± 0.05^b^	7.57 ± 0.01^b^	5.77 ± 0.01^e^
S7	18.37 ± 0.12^d^	35.67 ± 0.12^c^	31.84 ± 0.05^bc^	2.19 ± 0.05^b^	6.16 ± 0.06^f^	5.77 ± 0.01^e^
S8	17.30 ± 0.06^e^	39.74 ± 7.41^b^	31.03 ± 3.07^c^	1.77 ± 0.01^c^	5.05 ± 0.05^e^	5.11 ± 0.01^d^
S9	19.63 ± 0.22^c^	34.62 ± 1.48^c^	31.16 ± 1.04^c^	2.12 ± 0.01^b^	6.71 ± 0.16^e^	5.76 ± 0.06^e^
S10	13.27 ± 0.78^f^	41.15 ± 0.90^a^	31.09 ± 1.46^c^	1.75 ± 0.21^c^	7.17 ± 0.02^d^	5.57 ± 0.17^f^

Mean values in the same column within each substrate followed by different letters are significantly different (*p* < 0.05) according to Duncan’s multiple range tests. For S1-S10, see Table 1

### Mineral composition of the harvested fruiting bodies

The mineral composition of *P. ostreatus* harvested from all the different substrates was significantly (*P* < 0.05) affected by substrate formulations (Table 7). A significantly higher (*P* < 0.05) magnesium content of 1256.33 mg/kg was obtained from the oyster mushroom cultivated on the S3 substrate, while a lower magnesium content of 1141.02 mg/kg was recorded for the mushroom harvested from the S10 substrate. Similarly, mushrooms harvested from S5 substrate had a relatively higher potassium content (24021.20 mg/kg), followed by those cultivated on S4 substrate (23849.80 mg/kg) and S1 substrate (23401.01 mg/kg), whereas a lower potassium content of 21562.19 mg/kg was obtained from P. ostreatus grown on S9 substrate. Mushrooms cultivated on S1 substrate had significantly higher (*P* < 0.05) calcium (930.33 mg/kg), sodium (770.33 mg/kg), and iron (210.33 mg/kg) contents. Significantly higher and lower (*P* < 0.05) zinc content was recorded from S4 and S7 substrates, respectively (Table 7).

**Table 7:** Elemental content in *P. ostreatus* when grown on different substrates (mg/kg)

Substrates	Mg	K	Ca	Na	Zn	Fe
S1	1240 ± 2^ab^	23,401 ± 1^ab^	930 ± 1^a^	770 ± 1^a^	31 ± 0^abc^	210 ± 1^a^
S2	1216 ± 3^abc^	22,700 ± 5^abc^	854 ± 1^ab^	757 ± 1^a^	29 ± 1^bcd^	209 ± 1^a^
S3	1256 ± 2^a^	22,433 ± 1^bc^	715 ± 2^bc^	722 ± 1^ab^	27 ± 2^cde^	198 ± 2^a^
S4	1240 ± 1^ab^	23,849 ± 0^a^	616 ± 1^c^	540 ± 0^d^	34 ± 0^a^	180 ± 1^ab^
S5	1240 ± 2^ab^	24,021 ± 1^a^	687 ± 1^c^	751 ± 1^ab^	26 ± 0^de^	160 ± 1^ab^
S6	1191 ± 5^abc^	23,304 ± 2^ab^	742 ± 2^bc^	720 ± 2^ab^	26 ± 2^de^	156 ± 1^ab^
S7	1186 ± 4^abc^	22,690 ± 4^abc^	119 ± 2^e^	500 ± 3^d^	25 ± 1^e^	134 ± 1^c^
S8	1194 ± 8^abc^	22,754 ± 2^abc^	127 ± 1^e^	578 ± 0^cd^	31 ± 1^abc^	157 ± 1^ab^
S9	1166 ± 4^bc^	21,562 ± 1^c^	344 ± 1^d^	650 ± 1^bc^	28 ± 1^bcd^	192 ± 3^a^
S10	1141 ± 3^c^	22,358 ± 1^bc^	585 ± 1^c^	728 ± 1^ab^	33 ± 1^ab^	188 ± 2^ab^

Mean values in the same column within each substrates followed by different letters are significantly different (*p* < 0.05) according to Duncan’s multiple range tests. For S1-S10, see Table 1

## Discussion

### Nutritional composition of the substrates

*P. ostreatus* mushroom is proficient in decomposing lignocellulosic components, i.e., cellulose, hemicellulose, and lignin, as a carbon source for its mycelium growth and fruiting body development (Grimm and Wosten 2018), which makes them ideal for mushroom cultivation (Doroški et al. 2022). Nitrogen-rich compounds are used as supplements to mushroom cultivation substrates and result in higher mushroom yields with increased metabolic activities triggered by the presence of extra nitrogen according to the study of Rodriguez Estrada and Royse (2007), which contradicts the claim that higher nitrogen is the cause of mycelial growth interruption (Doroški et al. 2022). On the other hand, the addition of excess nitrogen-rich substrates may also lead to higher contamination risks by competitor microorganisms (Yildiz et al. 2002). The ideal nitrogen content of the substrate is reported to range between 0.5 and 2% Naraian et al. (2009), the value of which agreed with the results of the present study (Table 2). Moreover, the factors significant for mycelial growth, yield, and efficiency of mycelium production include the range of the C/N ratio, pH, and moisture contents (Nwanze et al. 2005). In addition, *P. ostreatus* requires inorganic compounds such as K, Ca, Mg, Na, and Zn to enhance yields and biological efficiency (Golian et al. 2021). Supplemented substrates with carbon or nitrogen compounds are important to shorten the spawn run time and increase the harvesting cycles of mushrooms (Ulziijargal et al. 2013; Cogorni et al. 2014).

### Spawn running, pinhead formation and fruiting body formation

The shortest spawn run time of 10 and 12 days was obtained from *P. ostreatus* grown on S7 substrate, followed by that of S6 substrate, in which the C: N ratio was 40.15 and 50.98% respectively (Table 2). The spawn run time recorded from the current study was faster (shorter) than that of Hoa et al. (2015), who reported that the spawn run period took three weeks for *Pleurotus ostreatus* grown on pure sugarcane bagasse substrate without any supplements. The current result is also in agreement with that of Musieba et al. (2012), who reported short colonization time and high mycelium density of mushroom cultivated on straw substrate. The current result is also in agreement with that of Atila (2017), who verified that safflower hay supplemented with wheat bran and Gypsum substrate supported fast colonization of 16.4 days for *P. djamor*. The finding of Albores et al. (2006) revealed that there was a positive correlation between the C/N ratio of substrate and mycelium growth rate.

Substrate with a lower C/N ratio supported fruiting body formation better than substrate with a high C/N ratio. This is supported by Naraian et al. (2009), who demonstrated that mycelium growth and primordial development of *Pleurotus florida* were dependent on the C/N ratio.

The result of the current study was similar to that of Yang et al. (2013) who showed that a higher C/N ratio favored mycelium growth, but a lower C/N ratio favored fruiting body growth, thereby reducing the final maturity to harvest time. Yang et al. (2013) also determined that oyster mushroom *P. ostreatus* grown on substrate with 80% cotton seed hull with C/*N* = 34.87 needed longer to complete colonization period than that cultivated on substrate of 80% rice straw and 80% wheat straw with C/*N* = 49.19 and C/*N* = 64.63, respectively. Hoa et al. (2015) reported that sugarcane bagasse provided 51.71% of C: N with early spawn run completion for *P. ostreatus* mushroom. This is due to the high nitrogen content, which commonly inhibits mushroom growth in excessive amounts (Bellettini et al. 2019). In addition, an excess nitrogen content in the growth substrates is known to delay the formation of the fruiting body and suggested that the C/N ratio that would favor the primordial and fruiting body formation is about 22–30:1 (Yang et al. 2013). Generally, the results of the current study confirmed that a higher C/N ratio favors mycelial growth and a lower C/N ratio favors fruiting body growth. According to the results of the current study, S1, S6, and S4 substrates were found to be richer in hemicellulose content compared to the others. Obodai et al. (2003) reported a negative relationship between yield and hemicellulose content of a substrate, which agrees with the results of the present study. However, there was a positive relationship between yield and hemicellulose during the first flushes (Table 2; Fig. 4), the results of which were in agreement with those of Atila (2017). Hemicellulose is mainly utilized during the active growth phase Bellettini et al. (2019), and the growing macro-fungi cannot immediately benefit from the lignin and cellulose content of a substrate Wang et al. (2006). Moreover, reported that nitrogen-rich materials increase the degradability of the lignocellulosic material to enhance the accessibility of essential nutrients. In the present study, mycelial growth may be accelerated by the increased utilization of lignocellulosic content such as hemicellulose, cellulose, and lignin in parallel with the nitrogen content. It appears reasonable to assume that the high hemicellulose content associated with the high nitrogen concentration exerts a positive effect on mycelial growth.

The fastest mycelial growth may or may not correspond with the appearance of pinhead formation (Table 5). Hence, the days of pinhead formation ranged between 2 and 6.67 days, the duration of which was shorter than that of Hoa et al. (2015) and Ahmad Zakil et al. (2022), who reported pinhead formation within 5.50–8 and 6–7 days. The fast growth and pinhead formation of mushrooms were attributed to the addition of supplements such as wheat bran and other nitrogen-rich substrates (Das and Mukherjee 2007). In this study, the addition of cow dung, horse manure, and chicken manure as nitrogen sources enhanced the mycelium growth of *P. ostreatus*.

The earliest first harvest (3 days) of *P. ostreatus* was recorded from S1 substrate (Table 6), the results of which were contrary to those of Mondal et al. (1970), who found that the first fruiting and harvesting of mushrooms cultivated on banana leaves and rice straw was 11.25 days. The result of the present study was similar to that of Atila (2017), who demonstrated the harvest date for *Pleurotus spp*. to be 4 to 5 days after pinheads’ formation. This difference is due to the difference in total C, total N, and C/N ratios of the substrate, which had more effects on mycelium growth, pinheads’ formation, and the development of the fruiting body.

### Cap diameter and stipe length

There were variations in stipe length and cap diameter of mushrooms cultivated on different substrates (Table 6). Hence, the stipe length of the products varied as shown in Table 5, which is very close to that of 100% rubber tree sawdust substrate, which gave a mean height of 9.12 to 7.75 cm for the best yield and the least yield, respectively Ahmad Zakil et al. (2020), while the cap diameter gave the highest value at 7.80 cm. The physical quality of oyster mushrooms depends on the length of stipe and it is suggested that in the case of yield, the larger the cap size, the higher the yield (Ahmed et al. 2013). Kortei et al. (2018) noted that the higher the stipe length and the smaller the mushroom cap diameter, the less desirable the quality of the marketable product. A similar result was reported by Yang et al. (2013) that the supplement of wheat bran and cotton seed hull to rice straw or wheat straw could shorten mushroom stipe length (3.1 cm) but increase mushroom cap diameter (9.2 cm), which confirmed the quality of mushrooms. Therefore, large-sized fruit bodies with shorter stipe are widely perceived to be of superior quality with high-ranking mushroom cultivation. Mushrooms with a relatively bigger pileus (cap), a higher thickness, and shorter but wider stipes are preferred for their marketable quality. This could be attained under optimum environmental conditions such as aeration, temperature, relative humidity, and substrate moisture holding capacity during the various growth stages (Vega et al. 2022). The results of the present study are in agreement with those of (Sánchez 2010; Onyango and Palapala 2011; Yang et al. 2013) indicated that locally available substrates with supplementation have been reported to boost the nutritional contents, yield, biological efficiency, mycelium growth, and quality of the fruiting bodies of the cultivated mushrooms.

### Weight, total yield, and biological efficiency

A higher yield of *P. ostreatus* was obtained from the first flush and gradually decreased during the subsequent flushes (Fig. 4). Significantly higher (*P* < 0.05) total yield and biological efficiency (744.20 g/bag and 148.80%) were recorded from the mushroom cultivated on S2 substrate (Fig. 4). The biological efficiency of *P. ostreatus* mushroom species in the present study was higher in comparison to the results of other studies Hoa et al. (2015) involved in the cultivation of mushrooms on corncob and sugarcane bagasse substrates that resulted in biological efficiencies of 66.08 and 65.65%, respectively. Khan et al. (2021) reported a higher total yield of 388.40 g/bag and biological efficiency of 77.68% from oyster mushrooms cultivated on a substrate comprising 50% office scrap paper and 50% poultry manure, the results of which were lower than those of the current study. In contrast, Bhattacharjya et al. (2013) observed that the biological efficiency of *P. ostreatus* ranged from 187.0 to 213.2%, the values of which were higher than those of the current study. The current results agree with that of Das and Mukherjee (2007), who indicated a biological efficiency of 139.0% of the mushroom cultivated on the combined substrates of rice straw and weed plants. This is significantly affected by the application of different substrates and supplements, like cow dung, to the main substrates (Kortei et al. 2018). Also, Siwulski et al. (2017) described that pure sawdust is not very effective for mushroom growth and produces poor products. However, sawdust mixed with different nutrient supplements had significant effects on mushroom yield and productivity.

The result of the present study shows that the C/N ratio of the substrate formulas used in this experiment is negatively correlated with the total yield and biological efficiency of *P. ostreatus* mushrooms. The current result was in agreement with that of Philippoussis (2009) who reported that there is a strong negative correlation between mushroom yield (mushroom number and BE) and the C/N ratio of the substrate. The optimum C/N ratio (40.42%) was found for biological yield and biological efficiency of the mushroom cultivated on S2 substrate, the values of which were in agreement with those of Atila (2017), who suggested an optimum C/N ratio of 35.7 and 40.6% *for P. ostreatus* and *P. florida*, respectively. The values were within the range of the C/N ratio of 32/1 ~ 150/1 reported by Chang and Miles (2004) to be effective for pinhead formation in *Pleurotus* species. On the other hand, Wang et al. (2006) showed that there was a positive correlation between biological efficiency and the degradation of cellulose and hemicellulose but reported a negative relationship between biological efficiency and lignin degradation. Hoa et al. (2015) indicated that organic substances rich in cellulose were one of the best substrates for the cultivation of oyster mushrooms. Substrates with high lignin content decreased the activity of cellulose, but less lignin would enhance enzyme activity and thus ensure higher mushroom yield and biological efficiency (Atila 2017).

### Nutritional composition of the harvested fruiting bodies

Protein insufficiency is one of the world’s most critical human dietary issues (Elkhateeb 2020). The protein content obtained in this study seems to be sufficient to solve this common problem. The high protein content of 13.27–21.53% obtained from mushrooms in this study could be due to the richness of carbon, nitrogen, and crude protein in the supplements added, as verified by the study of Odunmbaku, and Adenipekun (2018) which is essential for good health since protein helps in body growth, repair, and body tissue maintenance. Generally, the result obtained in this study is in agreement with that of Li et al. (2017), who cultivated *P. ostreatus* using cotton seed hull and perilla stalk with protein content ranging from 20.45 to 26.12%. Likewise, Cogorni et al. (2014) documented that the protein content of mushroom fruiting bodies cultivated on various substrates ranged from 20.33 to 25.33%. The results of which were similar to those of (Ashraf et al. 2013). The protein content obtained in this study is higher than the 5.2–10.85 reported by Das and Mukherjee (2007). Likewise, the protein and ash contents of the mushrooms obtained in the present study are comparable with that of Mintesnot et al. (2014), which may depend on the content of the growth substrates. Therefore, the protein content of the mushrooms depends on the selection and optimization of agro-industrial byproducts to enhance the dietary values of mushrooms.

The carbohydrate content achieved in this study indicated that mushrooms are a good source of energy, the result of which agrees with that of Li et al. (2017) and Tolera and Abera (2017), who reported carbohydrate contents ranging from 38.75 to 42%. The crude fiber content of *P. ostreatus* cultivated on different substrates ranged from 31.03 to 34.38%, which is higher than that of *P. ostreatus* cultivated on palm oil waste and sawdust supplemented with wheat and rice bran (Grimm et al. 2021; Elkanah et al. 2022). However, the fiber contents of the oyster mushroom are the same as those of *P. ostreatus* cultivated on sugarcane bagasse mixed with acacia sawdust (Adebayo et al. 2013). The current result is expected since sugarcane bagasse is a highly fibrous substrate and increases with proper formulation of the respective supplements.

The fat content obtained from *P. ostreatus* in this study is higher than that of (Onyeka and Okehie 2018; Grimm et al. 2021). Nevertheless, the low-fat content observed from *P. ostreatus* cultivated on all the substrates used in this study showed a lower caloric value (Elkanah et al. 2022). Low fat content makes mushrooms one of the most suitable dietary sources for patients with hypertensive problems. The moisture content of *P. ostreatus* cultivated on different substrates (6.14–8.82%) was generally lower, and the differences could be attributed to the drying level of mushroom samples (Adebayo et al. 2014). High water activities could increase microbial growth since they affect nutritional quality and chemical composition of *P. ostreatus* (Tolera and Abera 2017; Elkanah et al. 2022).

In the last three decades, human awareness of the promotion of good health through dietary intervention has increased, and eating edible mushrooms as sources of essential nutrients in diets is important for promoting human health (Oyetayo et al. 2013).

### Mineral composition of the harvested fruiting bodies

The mineral element composition of *P.ostreatus* harvested from all the substrates showed variation in concentration as reported elsewhere (Manzi et al. 2001; Mleczek et al. 2021). Potassium has an important role in metabolism Keskin et al. (2021) and in maintaining an osmotic balance between cells and the intestinal fluid in the animal system. According to Raman et al. (2020), the presence of potassium in *P. ostreatus* showed that oyster mushrooms would be good at lowering blood pressure, reducing the risk of osteoporosis, and maintaining bone health (Bilal et al. 2010). A mushroom-based diet is recommended for those suffering from hypertension and heart disease due to its high potassium-to-sodium ratio (Elkanah et al. 2022). According to the results of this study, the content of potassium in the harvested *P. ostreatus* cultivated on different substrates was variable, as suggested by the results of previous studies (Alaimo et al. 2018; Mleczek et al. 2020; Golian et al. 2021). Hence, based on these values, it was concluded that the obtained values are compatible with the literature data. As indicated in Table 6, sodium was the highest mineral element found in the mushroom fruiting bodies, with different concentrations based on different substrates (Zhou et al. 2023). According to (Cogorni et al. 2014; Gao et al. 2020) the sodium content in the samples of *P. ostreatus* in the stipe and in the cap showed different values, the results of which were consistent with the results of the present study.

There is a good balance between the high content of K and the low content of Na in mushrooms that can be implicated in curing high blood pressure (Manzi et al. 2001). The results of this study showed that *Pleurotus* mushroom species have nutritive potential endowed with medicinal importance. Calcium is an essential mineral and is important in the prevention and development of osteoporosis and in the formation of strong bones and teeth (Arnold et al. 2021). The calcium content determined in this study was generally higher compared to that of the previous studies (Adebayo et al. 2018; Zhou et al. 2023). On the contrary, lower concentrations of calcium were observed in the current study compared to the previous studies (Cogorni et al. 2014; Gao et al. 2020). Luckily, enough, the calcium content recorded in the current study is quite sufficient to provide the required dietary values. The zinc content recorded in this study was in the range of 25.26–34.61 mg/kg, the result of which was in agreement with that of Ahmad Zakil et al. (2022). Zinc is a necessary mineral in several enzymatic processes, such as DNA synthesis, material transitions in biological membranes, and the immune system (Keskin et al. 2021). Zinc requirements are 15 mg for adults and 3–5 mg for babies, indicating that mushrooms could contribute to human nutrition as a good source of Zn (Adebayo et al. 2014).

According to the results of the present study, mushroom fruiting bodies exhibited adequate concentrations of Fe when grown on different optimized substrates. Likewise, Riaz and Guerinot (2021) stated that Fe is one of the essential micronutrients that are required by plants and animals. Raman et al. (2020) demonstrated that about 90% of the bioavailability of Fe in the edible mushroom is easily absorbable, and its concentrations varied in different parts of the harvested mushrooms (Golian et al. 2021). In summary, the results of this study demonstrated a good nutrient profile of *P. ostreatus*, supporting the recommendation of lower energy intake and sodium and supplying enough protein content with low fat. Moreover, mushrooms can be supplied fresh as biomass dietary supplements.

## Conclusion

The results of this study suggest that S1 substrate containing 80% sugarcane bagasse, 12% chicken manure, and 8% cotton seed hull and S2 substrate could be economically viable substrates used to cultivate *P. ostreatus* mushroom species. The mushroom species *P. ostreatus* can be successfully cultivated on sugarcane bagasse supplemented with cow dung, cotton seed husk, and chicken manure, suggesting the possible use of these substrates in *P. ostreatus* commercial cultivation. Therefore, the productivity and dietary composition of the mushrooms are influenced by the nutritional composition of the formulated substrates. Thus, cotton seed hull, cow dung, and chicken manures could consequently be recommended as suitable supplements for enhancing (value-added) *P. ostreatus* growth, especially in terms of fighting malnutrition. The results of the current study indicated that most of the locally available agro-industrial byproducts could be diverted into mushroom production media by the government, food scientists, nutritionists, and other food supply stakeholders, aimed at economic gain, combating dietary deficiencies, and protecting the environment, accompanied by the production of human food of high nutritional and biological value.

## Data Availability

Data are accessible and can be sent to the concerned entity upon request.
